# Iron Homeostasis and *Trypanosoma brucei* Associated Immunopathogenicity Development: A Battle/Quest for Iron

**DOI:** 10.1155/2015/819389

**Published:** 2015-05-18

**Authors:** Benoit Stijlemans, Alain Beschin, Stefan Magez, Jo A. Van Ginderachter, Patrick De Baetselier

**Affiliations:** ^1^Laboratory of Cellular and Molecular Immunology, Vrije Universiteit Brussel (VUB), 1050 Brussels, Belgium; ^2^Department of Myeloid Cell Immunology, Vlaams Instituut voor Biotechnologie (VIB), 1050 Brussels, Belgium; ^3^Department of Structural Biology, Vlaams Instituut voor Biotechnologie (VIB), 1050 Brussels, Belgium

## Abstract

African trypanosomosis is a chronic debilitating disease affecting the health and economic well-being of developing countries. The immune response during African trypanosome infection consisting of a strong proinflammatory M1-type activation of the myeloid phagocyte system (MYPS) results in iron deprivation for these extracellular parasites. Yet, the persistence of M1-type MYPS activation causes the development of anemia (anemia of chronic disease, ACD) as a most prominent pathological parameter in the mammalian host, due to enhanced erythrophagocytosis and retention of iron within the MYPS thereby depriving iron for erythropoiesis. In this review we give an overview of how parasites acquire iron from the host and how iron modulation of the host MYPS affects trypanosomosis-associated anemia development. Finally, we also discuss different strategies at the level of both the host and the parasite that can/might be used to modulate iron availability during African trypanosome infections.

## 1. Introduction

Iron is a vital nutrient required by nearly every living organism ranging from archaea to eukaryotes. It is an essential cofactor present in heme groups and iron-sulphur clusters and impacts on a broad range of important biological/metabolic processes including host and pathogen cellular functions, erythropoiesis, and immunity. The capacity of iron to fluctuate between two oxidation states, ferrous (Fe^2+^) and ferric (Fe^3+^), makes it indispensable for many critical biological processes, including nucleic acid synthesis/DNA replication, lipid synthesis, protein translation, energy metabolism (cytochrome respiration), oxygen sensing/transport, and oxidant defense [1, 2]. Yet, the distinct oxidative state properties of iron which make iron indispensable also can contribute to toxicity to cells, because of its ability to promote the formation of damaging oxidative radicals. Indeed, the redox cycling of ferrous and ferric iron in the physiological presence of H_2_O_2_ in the cells results in the formation of reactive oxygen intermediates/free radicals (such as hydroxyl radicals) via the Fenton reaction which in turn can damage lipids, DNA, proteins, and other cellular components. Therefore, regulatory interactions between host iron homeostasis (quantity and subcellular location) and immune function are crucial, since both iron deficiency and iron excess can compromise cellular functions [3]. Access to iron is particularly important in the context of host-pathogen interactions. Indeed, when confronted with infection and inflammation the mammalian host reallocates its iron reservoirs in an effort to deprive invading intracellular or extracellular pathogens of iron [4, 5]. Thus, the control over iron homeostasis is a central battlefield in the course of an infectious disease [6, 7]. The host immune system can regulate iron availability for pathogens via activation of cytokines, cellular proteins/peptides, and hormones, hereby gaining control over pathogen proliferation and strengthening specific immune effector pathways, a strategy also termed “nutritional immunity” [6, 8, 9].

In this review we will give an overview of the role of iron homeostasis/modulation during African trypanosomosis, which is a chronic debilitating disease affecting the health and economic well-being of many people in developing countries [10–12]. It is caused by strictly extracellular/free-living flagellated unicellular parasites, which are etiologic agents of highly disabling and often fatal diseases of humans (i.e., Human African trypanosomosis, HAT) and livestock (i.e., Animal African trypanosomosis, AAT, or nagana). As recently reviewed [13], African trypanosomes produce a number of components that modulate the mammalian host immune response. In particular, they manipulate cells of the myeloid phagocytes system (MYPS) which includes myeloid cells of the mononuclear phagocytes system (MPS, i.e., macrophages, monocytes, and immature DCs) as well as granulocytes (neutrophils) [14] and thereby affect the capacity of the host to (i) control parasite growth (referred to as resistance to infection) and (ii) to limit tissue pathogenicity caused by the immune response mounted for resistance to infection (referred to as (trypano)tolerance to infection). Trypanotolerance is associated with the sequential induction of IFN-*γ* and MyD88-dependent M1-type myeloid cells (i.e., classically activated myeloid cells) producing TNF and/or NO which reduce the fitness of the parasite and ensure parasite control, followed by a switch to IL-10 dependent M2-type myeloid cells (i.e., alternatively activated myeloid cells) ensuring pathogenicity control [15]. In contrast, trypanosusceptibility is associated with a persistence of M1-type myeloid cells and an inability to switch to M2-type myeloid cells, which culminates in pathogenicity. In natural and experimental hosts, the control of the African trypanosome load, and thus resistance to infection, is less of a problem than the control of the immune response to mount tolerance to the disease. In this review, we will focus on how African trypanosomes acquire iron within the mammalian host and how iron modulation in host myeloid cells affects trypanosomosis-associated pathogenicity development, whereby anemia development is one of the most prominent parameters.

## 2. Iron Homeostasis/Acquisition in the African Trypanosome

Trypanosomatids comprise a large group of flagellated unicellular protozoa with a free-living and parasitic lifecycle. The 3 major human diseases caused by trypanosomatids are African trypanosomosis (sleeping sickness caused by* Trypanosoma brucei *sp.), South American trypanosomosis (Chagas' disease caused by* Trypanosoma cruzi*), and Leishmaniasis (caused by different species of* Leishmania*). With respect to the parasites that are the main focus of this review, members of the* T. brucei* complex are transmitted by tsetse flies of the genus* Glossina* spp., which are only present in equatorial Africa. These members can be further divided into the (i) human pathogens* T. b. gambiense* and* T. b. rhodesiense* and (ii) animal pathogens causing either nagana, which is mainly caused by* T. b. brucei*,* T. congolense,* and* T. vivax,* or surra (*T. evansi*) or dourin (*T. equiperdum*). Of note, the trypanosomes of equines (*T. equinum* and* T. equiperdum*) and of camels (*T. evansi*) are not transmitted by tsetse flies but by direct contact during copulation of horses by biting insects such as horse flies (tabanids). The two HAT causing parasites,* T. b. rhodesiense* and* T. b. gambiense*, do not only differ at the level of their geographical distribution; they also differ biologically, clinically, therapeutically, and epidemiologically and cause separate diseases. Indeed,* T. b. gambiense* infection (accounting for over 95% of cases) is found in West and Central Africa and progresses at a more indolent pace (up to 3 years) than that of* T. b. rhodesiense* (accounting for the remainder of cases) causing an acute, rapidly progressive infection (within 6 months) in eastern and southern Africa [11, 21]. Both infections are characterized by two stages, whereby in the first stage parasites are observed in the hemolymphatic system, producing fever, splenomegaly, adenopathies, and cardiac, neurological, and psychological disorders. In the second stage, trypanosomes are distributed in the central nervous system (CNS) leading to several sensory, motor, and psychic disorders and ending in death if untreated [11, 22]. For* gambiense* HAT, human beings are the main reservoir and the predominant mode of transmission is by tsetse flies although sexual and congenital transmission was also reported [23].* Rhodesiense *HAT, however, is a zoonosis, a “disease or infection naturally transmitted between vertebrate animals and humans,” whereby the transmission cycle thus involves mainly transmission between nonhuman reservoirs by tsetse flies, with occasional animal-tsetse-human transfer. Since* T. b. rhodesiense* and* T. b. gambiense* are morphologically identical and also resemble* T. b. brucei* (a subspecies causing nagana), the majority of research used* T. b. brucei* as a model. However, this might not always reflect what is happening in the case of HAT. Identification of both HAT species is based on specific molecular diagnosis markers, that is, the serum resistance-associated (SRA) gene which is restricted to* T. b. rhodesiense* parasites and the* T. b. gambiense* specific gene (TGSGP) which is restricted to* T. b. gambiense* [24, 25]. Alternatively,* T. b. gambiense* can also be diagnosed based on antibodies. According to WHO (World Health Organization, specialized agency of the United Nations serving as the directing and coordinating authority for international health matters and public health), HAT brings about 6 million people at risk of infection within the 36 affected African countries, most of them in rural areas of extreme poverty [22, 26]. Around 300,000 people are currently infected with trypanosomes and 10,000–40,000 of them die every year [11, 27]. Regarding AAT, the economic losses are estimated to be about US$ 1.2 billion per year due to major problems in agricultural and nutritional development of endemic areas [28]. Furthermore, the lack of prospect for vaccine development against African trypanosomosis is strengthened by (i) the fact that pharmaceutical companies are less prone to engage/invest in drug discovery/development of diseases that affect the poorest people, (ii) the political instability of the affected regions, (iii) the fact that wild animals function as reservoir of the parasite and therefore hamper the control of the disease, and (iv) the inappropriate use of the available drugs resulting in the emergence of drug resistance [29–31]. Nevertheless, so far chemotherapy remains the only therapeutic choice for these diseases, whereby they target unique organelles of trypanosomes such as glycosomes and the kinetoplast that are absent in the mammalian host or trypanosome metabolic pathways that differ from the host counterparts (carbohydrate metabolism, protein and lipid modifications, and programmed cell death) [32].

**Figure 1:**
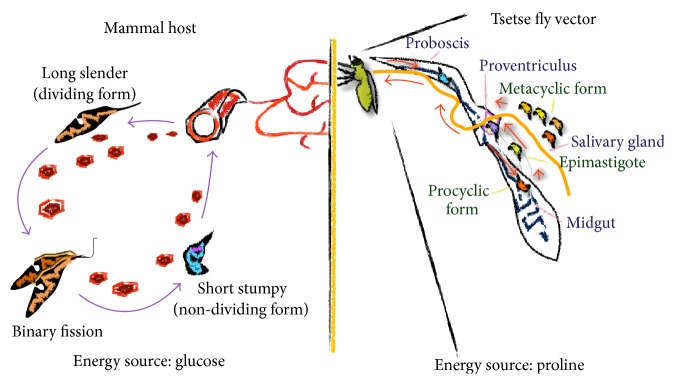
Lifecycle of African trypanosomes. Tsetse flies become infected following a blood meal taken from a trypanosome infected mammalian host. The parasites that are taken up reach the midgut together with the blood meal. Subsequently, only the short stumpy (non-dividing) forms of the parasite that are preadapted to the changed environment within the tsetse fly will be able to differentiate into procyclic forms. The multiplying procyclic forms colonize the ectoperitrophic space, after which they migrate to the salivary glands via the proventriculus lumen to move into the foregut and proboscis. During this migration, the procyclic forms in the fly differentiate into epimastigote forms that within the salivary glands attach to the epithelium and proliferate, giving rise to metacyclic forms which are preadapted for survival into the mammalian host [16, 17]. Upon a blood meal on a new host, the parasites will be inoculated and differentiate into a long slender (dividing) form. Within the mammalian host long slender forms multiply via binary fission, giving rise to a first peak of parasitemia. When the trypanosome population reaches a sufficiently high density, a quorum sensing-like mechanism elicits the differentiation of long slender forms into short stumpy forms that allow transmission following uptake by a new tsetse fly [18, 19]. During the entire lifecycle of trypanosomes, there is a continuous fight for iron acquisition at the level of both the parasites and the host. Image generated by Joar Pinto.

African trypanosomes have a strictly extracellular heteroxenous life cycle alternating between the intestine of the tsetse fly and the blood/tissues of the mammalian host (see Figure 1), whereby they exist as procyclic or trypomastigote forms, respectively [33]. Briefly, upon a bite of a trypanosome infected tsetse fly, metacyclic parasites are inoculated into the blood circulation of the mammalian host. The parasites immediately differentiate into long slender forms (dividing forms), which are adapted to survival in the glucose-rich and highly oxygenated blood of the host and multiply, thereby giving rise to a first parasitemia peak. Once a peak is reached, most likely due to quorum sensing [34], the long slender parasites differentiate into short stumpy forms (non-dividing forms), which are preadapted for survival in the tsetse fly vector (see Figure 1). Within the tsetse fly vector the parasites differentiate into procyclic forms which are adapted to survive in the proline-rich (carbon source) and low oxygenated environment. To this end, trypanosomes undergo important metabolic and morphological changes to adapt to the growth conditions imposed by the different hosts and environments they inhabit (Table 1) [33]. They have acquired elaborate mechanisms to adapt/survive in the different hosts such as fine tuning of energy metabolism, organelle reorganization, dedicated nutrient uptake, and biochemical and ultrastructural remodeling [35–38]. In particular, pathogenic trypanosomes have developed different mechanisms to guarantee iron supply from their host [39–41].

### 2.1. Iron Homeostasis/Acquisition by Bloodstream* T. brucei* Parasites

Trypanosomes in contrast to mammalian cells only require small amounts of iron [40], due to the fact that the bloodstream form lacks cytochromes and contains only 4 iron-dependent enzymes (aconitase, alternative oxidase, ribonucleotide reductase, and superoxide dismutase) [42–46]. Within the bloodstream of the mammalian host, trypanosomes scavenge iron via a high affinity receptor-mediated endocytosis of iron-bound transferrin (Tf), which is referred to as holo-Tf [47, 48]. This heterodimeric transferrin receptor (Tf-R), which is unable to discriminate between holo- and apo-Tf (iron-free Tf) [39, 49, 50], is encoded by the expression-site-associated genes (ESAG) 6 and 7, whereby the Tf-R protein is composed of one molecule of pESAG6 containing a COOH-terminal glycosylphosphatidylinositol (GPI) membrane anchor and one molecule of pESAG7 devoid of this modification [51, 52]. The heterodimer binds one molecule of Tf giving rise to a ternary complex. This low abundant glycoprotein (about 3000 molecules/cell) is located in the flagellar pocket and its expression is regulated by iron availability and posttranscriptional control mechanisms that does not involve the IRE/IRP1 (iron regulatory proteins/iron responsive elements) system typical for mammals [52, 53]. It was shown that iron starvation (using iron chelators or species specific Tf) leads to a 3–10-fold upregulation of Tf-R with a concomitant redistribution of the receptor from the flagellar pocket to the entire parasites' surface [52, 54]. Furthermore, it was shown that during chronic trypanosomosis in cattle the host Tf level is decreased, yet the bloodstream pathogens develop the ability to grow at very low iron concentrations by increasing their Tf-R expression levels thereby allowing higher Tf uptake [54, 55]. In addition,* T. b. brucei* has about 15 VSG expression sites (VSG-ES); only one is transcribed at a given time, while the others remain repressed, providing the expression of a particular combination of ESAGs in a mutually exclusive manner. The transcriptional activation/inactivation of genes in the VSG-ESs is a highly regulated mechanism, potentially allowing the parasite to quickly respond to any environment change. Furthermore, the different copies of ESAG6/7 sequences are highly polymorphic in regions corresponding to Tf binding sites, whereby small changes in the amino acids present in the surface exposed-loops drastically affect the affinity of the receptor for a given Tf, thereby contributing to an additional mechanism of trypanosomes to acquire iron and to permit their rapid adaptation in distinct hosts [56, 57]. Importantly, similar observations as for the* T. b. brucei* parasites with respect to the ESAG6/7 were observed for the* T. b. rhodesiense* and* T. b. gambiense* parasites [58, 59]. This Tf-R polymorphism which allows selecting for high affinity Tf-Rs together with the rapid recycling of Tf-R and gene-specific activation events enables trypanosomes to efficiently compete for limiting substrate and withstand iron deprivation until a new set of Tf-R is expressed [55, 60, 61]. The Tf-R which has an affinity 50–1000 nM for mouse/human Tf is exclusively present in the mammalian bloodstream stage form of the parasite and its structural organization differs completely from the mammal counterpart [58, 62]. Following binding of iron-bound Tf, the Tf-R is endocytosed in a temperature- and energy-dependent manner, which involves proteins like dynamin, epsin, the adaptor AP-2, the small GTPase TbRab5A, *β*-adaptin, and clathrin [63–65]. In addition, it was suggested that the phosphatidylinositol-3 kinase (PI-3K) TbVPS34 also plays a role in Tf trafficking, possibly downstream of TbRAb5A GTPase [66]. Upon cleavage of the intracellular GPIs by the GPI-phospholipase C (GPI-PLC) expressed in bloodstream* T. brucei*, producing diacylglycerol (DAG) and inositolphosphoglycan, DAG receptors are activated. Subsequently, the DAG signaling pathway is activated that depends on protein tyrosine kinase (PTK) for the activation of proteins in the endocytic system by the phosphorylation of clathrin, actin, adaptins, and other components of this machinery [67]. It was suggested that DAG stimulation of Tf uptake may contribute to parasite virulence by aiding* T. brucei* to acquire sufficient amounts of Tf (i.e., iron) to sustain its extracellular existence and compete with host cells for Tf in the blood [67]. Due to acidification within the endosomes, the iron is released from Tf, while the ESAG6/7 Tf-R complex loses affinity for apotransferrin [68]. Subsequently, while (apo)transferrin is delivered to the lysosome and proteolytically cleaved via a* T. brucei* cathepsin B-like protease TbcatB [53, 69, 70] and degraded fragments are exported/exocytosed from the cell via TbRAb11 positive recycling vesicles [71], the ESAG6/7 Tf-R is recycled back to the flagellar pocket via TbRAb11 positive recycling vesicles [72]. This is in contrast to the uptake of mammalian iron-bound Tf where the entire Tf-TfR complex is recycled to the cell surface (see later). How iron gets from the endolysosomal system to the cytoplasm is under investigation, but this involves possibly divalent metal ion transporters as in the mammalian host (see later). Given that Tf-bound iron (Fe^3+^) is practically insoluble at physiological pH and temperature, it must first be reduced to Fe^2+^, most likely through a ferric reductase, in order to be exported from the endolysosomal system into the cytoplasm. Interestingly, in the* T. brucei *genome, two putative ferric reductases have been found, a cytochrome b561-type (Tb927.6.3320) and an NADPH-dependent flavoprotein (Tb11.02.1990), which could act in cooperation with some divalent putative cation transporters [39]. In this context, recently, Taylor et al. [73] showed that a* T. brucei* Mucolipin-like protein TbMLP, orthologous to the mammalian endolysosomal cation channel Mucolipin 1, is involved in import of iron into the cytosol of African trypanosomes. It is expressed in both bloodstream and insect stages of the parasite and is confined to the endocytic system, with the highest expression being found in the p67-positive compartment (i.e., the lysosome). Yet, they also indicate that even when TbMLP expression is greatly reduced, there is sufficient iron import. Thus, an alternative mechanism to provide the parasite with an adequate supply of cytosolic iron needed for synthesis of iron-containing proteins is not excluded. For instance, it is suggested that intracellular iron is not homogeneously distributed. Excess iron is presumably transported to a storage compartment from which it can be released if cytosolic iron falls under a certain level. The signal for Tf-R upregulation could, therefore, come from a decrease in cytosolic iron. How the trypanosome monitors cytosolic iron is not known; however, cytosolic aconitase, the iron sensor in mammalian cells, is not involved in* T. brucei* iron sensing.

Besides the Tf-R,* T. brucei* parasites can also acquire iron via the uptake of iron-binding Lactoferrin (LF), a member of the Tf family protein found in most biological fluids of mammals and secondary granules of polymorphonuclear cells (PMN) [74]. One of the receptors involved in LF binding was glyceraldehyde-3-phosphate dehydrogenase (GAPDH) [74], an enzyme typically involved in catalyzing several steps in glycolysis (i.e., breakdown of glucose for energy and carbon molecules) and also found to be involved in several nonmetabolic processes including transcription activation and initiation of apoptosis. It is considered to be a member of the moonlighting or multifunctional proteins [75]. Iron can also be acquired via the uptake of heme, which consists of a cyclic tetrapyrrole ring (protoporphyrin IX) that coordinates an iron atom which can adopt Fe^3+^ or Fe^2+^ oxidation states. This essential cofactor for proteins is involved in oxygen transport and storage (hemoglobin and myoglobin), mitochondrial electron transport (complexes II–IV), steroid metabolism (cytochromes), signal transduction (nitric oxide synthase), and transcription and regulation of antioxidant-defense enzymes (e.g., superoxide dismutase, catalase, and peroxiredoxins). Heme is also a regulatory molecule involved in gene transcription/translation [76, 77]. Given that trypanosomes are auxotrophic for heme [78], they have adapted their heme-dependent metabolic pathways (biosynthesis of sterols and polyunsaturated fatty acids, respiration, oxidative stress response, and detoxification) to fluctuations in nutritional availability across their life cycle [77]. Host heme cannot diffuse through the parasites' membrane due to the fact that it contains cationic carboxylate side chains. Thus, trypanosomes have evolved high affinity heme-binding proteins on their cell surface. To this end, the parasites express an haptoglobin-hemoglobin receptor (HpHbR), which is linked to the plasma membrane through a C-terminal GPI anchor and localized in the flagellar pocket of bloodstream parasites only [79, 80]. HpHbR also plays a central role in determining whether humans can be infected by trypanosomes [79]. Most species of trypanosomes, such as* T. b. brucei*, are unable to infect humans due to the trypanolytic serum protein apolipoprotein-L1 (APOL1) delivered via two trypanosome lytic factors (TLF-1 and TLF-2). Binding of TLF1 to the HpHbR results in endocytosis and lysosomal localization of the toxin, apoL-I, and subsequent death of the parasite. However,* T. brucei rhodesiense* and* T. brucei gambiense* have managed to resist this lysis mechanism. Indeed,* T. brucei rhodesiense* expresses the ApoL1 neutralizing serum resistance-associated (SRA) protein, which is a truncated version of the variant surface glycoprotein (VSG), which binds to APOL1 in the lysosome and hence prevents lysis [81].* T. brucei gambiense* has besides a single highly conserved amino acid mutation in the TbgHpHbR thereby ablating high affinity TLF-1 binding and subsequent endocytosis, also a* T. b. gambiense* specific gene,* TgsGP*, which is also a truncated version of VSG [82]. With respect to heme transport and distribution in trypanosomes, these mechanisms remain so far elusive. Moreover, since African trypanosomes lack heme oxygenase and ferrochelatase required for heme catabolism and extraction of iron, the simplest explanation could be that scavenged heme is incorporated directly into heme-proteins which are distributed throughout different subcellular compartments without intermediate steps [78].

### 2.2. Iron Homeostasis/Acquisition by Insect Stage* T. brucei* Parasites

Switching from the mammalian host nutrient rich environment to the tsetse fly vector nutrient-poor environment requires a change in the energy metabolism of the parasites in order to survive. In contrast to the bloodstream form stage of trypanosomes where iron uptake occurs mainly via a Tf-R mediated mechanism, the iron uptake mechanisms in the procyclic insect stage form of* T. brucei* are less characterized. It is accepted that procyclic forms efficiently take up iron from ferric complexes via a reductive mechanism [83]. Surprisingly, although the insect stage requires more iron, the rate of endocytosis is greatly reduced compared to the bloodstream stage [35, 84]. This can be explained by the activation of a mitochondrial respiratory chain for energy metabolism in the insect life stage of the parasite [43, 85]; the bloodstream form of* T. brucei* shows a rudimentary mitochondrion but through the activity of an alternative oxidase and well-developed glycosomes depends on glycolysis for its energetic metabolism while the procyclic form of* T. brucei *presents a well-developed mitochondrion and low glycosomal activity. Given that the procyclic form (i) requires active mitochondria which are one of the most important heme-protein containing organelles and (ii) resides in the midgut of the tsetse fly where hemoglobin digestion following a blood meal results in massive release of free heme, uptake of heme or heme-containing proteins might be more important in this stage [86, 87]. The* T. brucei* vacuole protein sorting 41 (TbVPS41) plays an important role in the intracellular iron utilization system as well as the maintenance of normal cellular morphology in the procyclic form of the parasite [88]. In addition, it was shown that iron-sulphur (FeS) cluster proteins involved in a variety of cellular processes including electron transport and gene expression, such as the Rieske protein and cytochrome c reductase (complex III), in which iron atoms in different activation states are coordinated with inorganic sulphur in addition to cystein thiol groups, contribute to incorporation of heme into apo/heme-proteins [78, 89], which are involved in essential metabolic pathways like biosynthesis of sterols and polyunsaturated fatty acids (PUFAs) carried out in the endoplasmic reticulum (ER) and respiratory complexes in the mitochondrion [77].

## 3. Iron Homeostasis/Acquisition in the Mammalian Host during Steady-State Situation

Given that African trypanosomes multiply in the mammalian bloodstream as extracellular parasites, they continuously depend on host nutrient supply but are also confronted with the host's immune system. Hereby, the mammalian host immune system regulates iron availability for pathogens to gain control over pathogen proliferation and to strengthen the specific immune effector mechanisms via cytokines, cellular proteins/peptides, and hormones. Over the years, the molecular mechanisms involved in the regulation of iron homeostasis in the mammalian host have become increasingly clear [90, 91]. These mechanisms are summarized in the next paragraph since they provide the conceptual framework for our investigations into the role of iron in host-African trypanosome interactions.

**Figure 2:**
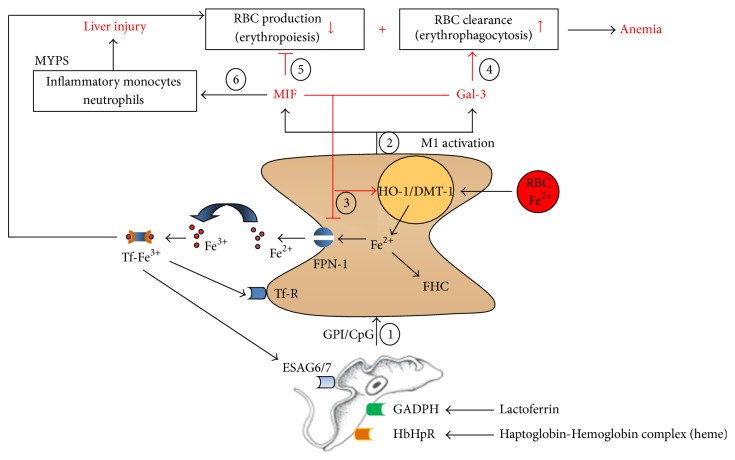
Iron modulation and pathogenicity development during* T. brucei* infection. Trypanosomes are equipped with different molecules to acquire iron from the mammalian host, namely, via their ESAG6/7 Tf-R (involved in Tf uptake), GADPH Lf-R (involved in Lf uptake), and the HbHpR (involved in heme/hemoglobin uptake). Parasites release molecules like the GPI-anchor or CpG-DNA to modulate/activate the host MYPS for their own benefit (1). Most of these molecules released by death or phagocytosed parasites trigger the release of proinflammatory molecules by M1-type myeloid cells, including Gal-3 and MIF (2). Both molecules stimulate iron-retention by inducing expression of HO-1, DMT-1, and FHC and by decreasing expression of FPN-1 within M1-type MYPS cells (3). Gal-3 by stimulating erythrophagocytosis (4) and MIF by suppressing erythropoiesis (5) contribute to anemia development. Due to their antiapoptotic effect, Gal-3 and MIF favor the persistence of the pathogenic M1-type MYPS. Moreover, MIF contributes to the recruitment of other pathogenic myeloid cells such as monocytes and neutrophils and further fuels the development of liver injury (6). GPI: glycosylphosphatidylinositol; Gal-3: galectin-3; MIF: macrophage migration inhibitory factor; Tf: transferrin; Tf-R: transferrin-receptor; Lf: lactoferrin; Lf-R: lactoferrin-receptor; HO-1: heme oxygenase 1; DMT-1: Divalent metal ion transporter 1; FPN-1: ferroportin-1; ESAG6/7 Tf-R: expression-site-associated genes (ESAG) 6 and 7; GAPDH: glyceraldehyde-3-phosphate dehydrogenase; HpHbR: haptoglobin-hemoglobin receptor; MYPS: myeloid phagocyte system. Figure adapted from [20].

Free iron or heme-bound iron uptake/absorption occurs primarily in the duodenum across the apical mucosa of duodenal epithelial cells, whereby intestinal heme iron uptake occurs through the interaction with the heme carrier protein (HCP1). The iron in the heme is then released within the enterocytes via the action of the heme catabolizing enzyme heme oxygenase (HO-1) and the dietary ferric iron (Fe^3+^) is reduced to the ferrous (Fe^2+^) state by ferric reductases at the level of the enterocytes [92, 93]. Subsequently, ferrous iron is transported into the cell via the divalent metal ion transporter 1 (DMT1, Nramp2 (natural resistance-associated macrophages protein)/solute carrier family 11, member 2 (SLC11A2)), after which it can be used for cellular processes or stored intracellularly by ferritin or exported from the cell at the basolateral membrane to the plasma via the sole iron exporter ferroportin-1 (FPN-1, SLC40A1), depending on the hosts' requirements for iron. Associated with ferroportin is the enzyme hephaestin (a copper-containing ferroxidase with homology to ceruloplasmin (see later)) which oxidizes the ferrous form back to the ferric form. Once in the circulation, liver secreted Tf will bind one or two ferric iron molecules and transport iron in the serum and extravascular spaces where it serves as a source of iron for cells and tissues that are perfused by the systemic circulation, including liver, heart, muscle, kidney, and bone marrow. Hereby, two main factors determine iron absorption; (i) the amount of iron present in body stores and (ii) the need of iron for hematopoiesis/erythropoiesis [94]. Important to mention is that the amount of iron absorbed via alimentation is insufficient to meet the daily physiologic needs of the body. Therefore, the bulk of iron needed for homeostasis (mainly for red blood cell (RBC) production) is provided by the MYPS, more specifically the myeloid cells, that recycles senescent RBCs at the level of the liver, spleen, and bone marrow via erythrophagocytosis and allows their iron reutilization [95] (see Figure 2). The majority of the body iron in mammals is sequestered intracellularly and complexed within the heme moiety of hemoglobin inside RBCs. Catabolism of RBCs results in heme release which in turn is further processed via heme-oxygenase 1 (HO-1) to give rise to equal amounts of iron, biliverdin, and carbon monoxide [96]. Once iron is released from heme it follows the same pathway as iron released from the holo-Tf/Tf-R complex, whereby it is either stored in the cell by ferritin or exported via ferroportin-1 (see further). Under physiological conditions, iron recycling by macrophages accounts for approximately 95% of the daily needs of iron for erythropoiesis and other physiological processes [97, 98]. Given that there is no regulated pathway to excrete iron from the body, the iron balance is primarily preserved by the regulation of iron absorption from the duodenum and iron recycling from myeloid cells and other tissue stores (primarily within hepatocytes) [99].

Iron exported from enterocytes or cells from the MYPS is bound with high affinity to transferrin (Tf), a glycoprotein produced in the liver and able to bind one or two iron molecules. Tf has a dual function (i) limiting iron-catalyzed free radical production and (ii) facilitating iron transport to all cells within the host that requires iron [100]. Alternatively, circulating copper-carrying ceruloplasmin can also participate in iron transport [101]. Under steady state situations iron-bound Tf (holotransferrin) is taken up via endocytosis by cells that express the Tf receptor (Tf-R) in order to fulfill normal metabolism, DNA synthesis, and RBC production. Important to mention is that the mammalian Tf-R is a homodimeric transmembrane glycoprotein consisting of two identical monomers joined together by two disulphide bonds and composed of a short cytoplasmic NH2-terminal cytoplasmic region (residues 1–67), a single transmembrane pass (residues 68–88), and a large extracellular portion (residues 89–760) containing the Tf binding region. It can bind two molecules of Tf with an affinity of 10 nM [102–104]. There are two isoforms, Tf-R1 (expressed by all nucleated cells) and TfR-2 (restricted to hepatocytes and immature erythroid cells), present within the mammalian host that are different from the trypanosomal Tf-R. In addition, Tf-R1 levels are regulated by cellular iron levels while Tf-R2 levels are regulated by Tf saturation and reported to bind diferric Tf although with 25 times lower affinity than Tf-R1. Also at the level of the kidney, some Tf which is an essential growth factor in the development of kidney and differentiation of tubules normally enters glomerular filtrate, but it is mainly retrieved by specific receptor-mediated uptake in the kidney tubular system. The Tf-R is expressed on the apical membrane of proximal tubule and collecting duct cells. Also, in the proximal tubule, the cubilin receptor, which is highly expressed on the apical membrane of kidney proximal tubules, is thought to mediate uptake of Tf [105]. Of note, similar to trypanosomes, mammals are able to bind Tf via GADPH, albeit with lower affinity [106, 107].

Internalization of the iron-Tf-R complexes is initiated following receptor phosphorylation by protein kinase-C (PKC). Following uptake of holo-Tf, the lower pH of the endosome/phagolysosome triggers the release of iron and recycling of the apo-Tf/Tf-R complex to the cell surface, whereby the apo-Tf is released due to the neutral pH. Subsequently, ferric iron is reduced and transported into the cytoplasm by Nramp1 (a divalent metal transporter homologous to DMT1 expressed at the phagolysosomal membrane) where its faith depends on the cellular needs: (i) stored in ferritin (a large globular protein-complex able to bind up to 4500 iron molecules) or (ii) exported/released back into circulation via the basolateral transmembrane iron transporter ferroportin (FPN1/SLC40A1) followed by oxidization of the ferrous form back to the ferric form via ceruloplasmin. As mentioned before, to meet the cellular needs of the body and to prevent cellular damage by iron, the amount of iron in the body must be tightly regulated. Hereby, the liver is a central regulator of systemic iron homeostasis through secretion of the 25-amino-acid peptide hormone hepcidin mainly by hepatocytes [99, 108, 109]. Furthermore, the hepcidin production is also regulated via different triggers, whereby iron levels, proinflammatory cytokines (such as IL-1, IL-6, and IL-22), TLR activation, or endoplasmatic reticulum stress stimulate its production, whereas erythropoiesis, anemia, hypoxia, hormones (estrogen), and growth factors (epidermal growth factor, hepatocyte growth factor) decrease its production [94, 110–114]. Of note, neutrophils and myeloid cells can also synthesize minute amounts of hepcidin in response to infectious agents, thereby allowing the modulation of iron availability in an autocrine fashion at the infectious focus [115, 116]. Following binding of hepcidin to the principal iron exporter FPN-1 at the cell surface, this latter is internalized and subsequently lysosomally degraded [117]. As a result, the export of iron is blocked, and iron is sequestrated at the level of the enterocytes, myeloid cells, and hepatocytes [109]. As mentioned before, limiting the iron availability for extracellular pathogens is considered to be a defense mechanism of the body, yet, reducing the levels of circulating iron can also culminate in anemia development [1, 118].

## 4. Iron Homeostasis/Acquisition in the Mammalian Host during African Trypanosome Infection

In livestock populations, anemia is considered the major cause of death during African trypanosomosis, and the capacity to limit anemia is critical in determining trypanotolerance [119]. The occurrence in infected cattle of hyper activated M1-type myeloid cells and massive erythrophagocytosis by tissue-associated MYPS cells as well as a modulated iron homeostasis suggests that these factors can be major causes of anemia [120, 121].

To unravel the mechanisms underlying African trypanosomosis-elicited anemia development, murine models (focusing on* T. brucei* trypanosomosis) exhibiting different degrees of anemia development were scrutinized. Although these mouse models show limitations, they have contributed significantly to our current understanding of trypanosomosis-associated anemia development [13, 20, 122]. In* T. brucei*-infected mice, anemia level does not correlate with parasitemia levels, antibody and T-cell responses, or survival time [119, 123], similar to bovine trypanosomosis [119, 124], suggesting that anemia is a consequence of the host immune response rather than of a direct influence of parasite products on RBC viability.

Anemia development during the course of experimental* T. brucei* infection can be divided into two phases (reviewed in [20, 125]): (i) an acute phase whereby M1-activated myeloid cells (i.e., classically activated myeloid cells) eliminate/phagocytose RBCs mainly in the liver and the spleen, resulting in activation of pathways that govern iron homeostasis and (ii) a short-lived and partial recovery of RBC levels, which is most likely due to extramedullar erythropoiesis (at the level of the spleen and liver) in response to the acute anemia in an attempt to restore RBC numbers, followed by a chronic and progressive phase. During this phase, persistence of M1-type cells of the MYPS reduces iron bioavailability by retaining iron in storage sites within the MYPS, thereby diverting iron from erythropoiesis. In this chronic phase, the enhanced uptake of both RBCs and iron-containing compounds is maintained, which aggravates anemia development [125]. The upregulation in whole tissue and cells of the MYPS of molecules involved in import (HO-1/DMT-1) and storage (FHC) of iron and downregulation of the cellular iron export regulator (FPN-1) provides additional evidence for an augmented liver and spleen iron-metabolism and accelerated senescence of RBCs during African trypanosome infection in trypanosusceptible animals (Figure 2) [125, 126]. Moreover, erythropoiesis was shown to be suppressed during the course of trypanosome infections [126–128]. It should also be remarked that iron accumulation in M1-type cells of the MYPS can also contribute to oxidative stress and NF-*κ*B activation [129–131], thereby contributing to liver pathogenicity occurring during African trypanosomosis [132, 133]. Thus, although limiting iron availability for pathogens during the acute phase of infection as an “iron withholding strategy” can prevent parasite development and benefit the host [1, 2], persistence of this response in the chronic phase of infection can disadvantage the host. Indeed, iron sequestration by MYPS cells can fuel their M1-type activation status and limit iron availability for erythropoiesis [134], thereby contributing to persistence of anemia.

## 5. Immune Modulation of Iron Homeostasis in the Mammalian Host during African Trypanosome Infection

The anemia induced during African trypanosomosis in mice, which is characterized by an imbalance between erythrophagocytosis and erythropoiesis and by an altered iron recycling and sequestration by MYPS cells, relates to the so-called anemia of chronic disease (ACD) [20, 90]. Studies over the past years aiming at unraveling the underlying mechanisms involved in anemia development during* T. brucei* infection have shown the following:

- IFN-*γ*, TNF, TNF-R2, and MyD88-deficient mice exhibit lower anemia levels as compared to control wild-type C57Black/6 mice [135–138]. Accumulated data suggest that the MyD88-dependent activation of the innate immune response results in the induction of IFN-*γ* by T cells. Subsequently, TNF production is triggered, whereby TNF-R2 signaling plays a key role in the induction of pathogenicity. In fact, the increased ratio of TNF over its soluble TNF-R2, not TNF levels per se, relates to the occurrence of infection-associated anemia [136].
- The activation state of cells of the MYPS plays a detrimental role in pathogenicity development, whereby M1-type MYPS cells (classically activated myeloid cells) in trypanosusceptible animals (exhibiting severe anemia/ACD) triggered via IFN-*γ* and/or molecules acting via TLR signaling promote the production of proinflammatory cytokines (such as TNF and IL-6) and the sequestration of iron. In contrast, M2-type MYPS cells (alternatively activated myeloid cells) in trypanotolerant animals (exhibiting reduced anemia/ACD) induced via IL-10 or IL4/IL13 promote induction of anti-inflammatory cytokines like IL-10 (crucial for dampening the pathogenic effects of proinflammatory cytokines) and export of iron [139–141]. Moreover, trypanotolerant animals in contrast to trypanosusceptible animals exhibit a restored iron homeostasis and increased iron availability for erythropoiesis [127].
- A comparison between trypanosusceptible and trypanotolerant animals allowed identification of two host-derived pleotropic molecules, Galectin-3 and macrophage migration inhibitory factor (MIF), which are strongly upregulated in* T. brucei* infected mice. Galectin-3 (Gal-3), a lectin contributing to the onset and persistence of type-1 inflammatory responses and phagocytosis, was shown to be involved in trypanosomosis-associated anemia development [142]. Hereby,* T. brucei *infected galectin-3 deficient mice exhibit greatly reduced anemia levels coinciding with a restored iron homeostasis and an increased IL-10 level that in turn leads to reduced liver destruction. With respect to MIF, we recently showed that MIF deficient animals feature limited anemia, which coincides with a reduced proinflammatory immune response, an increased iron bioavailability, improved erythropoiesis, reduced RBC clearance, and increased IL-10 levels associated with decreased liver injury during the chronic phase of infection [143]. In addition, neutrophil-derived MIF contributed more than monocyte-derived MIF to pathogenicity during* T. brucei* infection. Collectively, these results suggest that the M1-type MYPS cell associated molecules galectin-3 and MIF both promote the most prominent pathological features of experimental trypanosome infections (anemia and liver injury) (Figure 2).

Besides identifying host-derived molecules involved in triggering/maintaining M1 cells and consequently being detrimental in anemia development/iron sequestration, the identification of parasites-derived molecules triggering M1-type MYPS cell development could have potential therapeutic applications. In this context, the trypanosomal GPI-anchor was shown to be the most potent parasite-derived molecule able to trigger M1-type myeloid cells [144, 145]. Moreover, a GPI-based intervention strategy was found to alleviate trypanosomosis-associated anemia development by lowering the proinflammatory cytokine production (including TNF, MIF, and Gal-3) and increasing IL-10 production [146]. In addition, this treatment strategy restores iron homeostasis at the level of the liver (increased iron export and reduced storage) and increases erythropoiesis in the bone marrow and the spleen [127]. This suggests that reprogramming MYPS cells towards an anti-inflammatory state can be a promising tool to alleviate ACD, normalize iron homeostasis, and restore erythropoiesis. Although most trypanosomes cannot be considered natural pathogens for rodents, murine models represent excellent tools to study trypanosome biology and their interaction with the mammalian immune response. Furthermore, research so far on HAT patients suggests that TNF is also involved in immunopathogenesis of late stage African trypanosomosis and that IL10 plays an important regulatory role in the disease [122]. In addition, chemokines documented to contribute to pathogenesis such as CCL2 and CXCL10 within the murine model were also found in cerebral spin fluid (CSF) of HAT patients [147–149]. Yet, more additional work should be devoted to determine whether results from the murine model reflect observations in HAT patients.

## 6. Blocking Iron Uptake/Homeostasis at the Level of the African Trypanosome

Iron deprivation may represent a new strategy for treatment of African trypanosomosis. However, so far there is only a limited amount of drugs documented to block trypanosomal iron uptake or metabolism. The iron chelator/siderophore deferoxamine/desferoxamine (DFO) produced by* Streptomyces pilosus* has been developed into the drug Desferal which is used for the treatment of acute iron poisoning and chronic iron-overload. DFO also stops* T. brucei* parasite growth* in vitro* [150, 151]. This compound does not inhibit iron-containing enzymes directly but acts by chelating cellular iron, thus compromising the activity of Fe^3+^-containing enzymes such as ribonucleotide reductase, which is involved in DNA synthesis, and thereby preventing its incorporation into newly synthesized apoproteins. In their quest to identify novel iron-chelating molecules, the group of Merschjohann and Steverding [150] has shown that although most iron chelators tested so far also display some cytotoxicity to mammalian cells, only lipophilic iron-chelating agents represent potential as novel antitrypanosomal drugs. However, so far there are no published studies of the effect of direct iron chelation on* T. brucei *infection in animal models.

## 7. General Conclusions/Perspectives

Given that all organisms on earth depend on iron to fulfill vital cellular functions, there is a continuous quest of both pathogen and host to acquire this primordial metal. As far as parasites like African trypanosomes are concerned, their complex lifecycle alternating between the tsetse fly vector and the mammalian host adds an additional problem in the struggle for the supply of this metal.

At the level of the mammalian host, the concept that polarization of cells of the MYPS into distinct M1-type or M2-type activation states contributes to trypanosusceptibility or tolerance, respectively, suggests that reprogramming of MYPS cells may provide new therapeutical modalities in the treatment of infection-associated pathogenicity development [15]. However, additional research is required to dissect the exact contribution of the different liver and spleen associated MYPS cell subsets (Ly6c+ and Ly6c− monocytes, resident and Ly6c+ monocyte-derived macrophages, granulocytes, and dendritic cells) in erythrophagocytosis or modulation of iron homeostasis and to Gal-3 or MIF production. Recently, a mammalian MIF homologue D-dopachrome tautomerase (D-DT or MIF2) has been identified [152], but its role during African trypanosomosis-associated pathogenicity development (anemia) remains to be investigated.

At the level of the parasites, strategies able to block parasites' endocytosis of iron-containing molecules can form alternative approaches to control parasite development and survival [153–156]. Hereby, blockage of uptake of iron or of iron-containing compounds via antagonists or antibodies is promising. For instance, the specificity of the trypanosomal (ESAG6/7) Tf-R makes it a potential target to deliver toxic molecules inside the parasite [47]. In this context, a therapy based on nanobodies (Nbs) which are camelid derived single-domain antibody fragments [157] was found efficient in targeting drugs to African trypanosomes [158]. Also, Nbs able to block endocytic capacity of the parasite were found to block transferrin uptake thereby killing the trypanosome [156]. Moreover, a functional* T. brucei* Tf-R was expressed in insect cells which could be helpful in crystallographic studies to determine the structure and characterize the interface between Tf and its receptor, which could lead to a new approach to combat infection [159]. Alternatively, the GPI-biosynthesis pathway which is crucial in parasites' viability may represent another therapeutic approach for trypanosomosis [160]. In addition, future efforts should also aim at improving the selectivity of iron chelators, for instance, by utilizing the wealth of information currently being generated in the development of cell-permeable iron chelators as cancer chemotherapeutic agents [161]. Moreover, iron chelators could be of interest for combination therapy with existing antitrypanosome drugs like, for instance, DFMO (eflornithine) [31]. Indeed, eflornithine (Ornidyl), by blocking polyamine (spermidine) biosynthesis and consequently by preventing the production of the antioxidant trypanothione synthesis by the African trypanosome [162], may cause oxidative stress by increasing the level of iron available for the Fenton reaction [163, 164]. However, it should be taken into consideration that the administration of iron chelators is not exempt from risks for the host due to their iron withholding activity which may lead to anemia. African trypanosomes are heme auxotrophs and are dependent on specialized transporters to import heme [165, 166]. Drug targeting of this transport pathway may be more valuable to target the insect stage of African trypanosomes where the heme import is more important than for the mammalian bloodstream stage of the parasite.

Despite the progresses achieved in the last years, more studies on the role of iron in the parasite development and on modulation of iron metabolism in infected hosts are required before translation of this knowledge into effective treatments.

## Figures and Tables

**Table 1:** Overview of the metabolic changes and differences in energy/iron source used by African trypanosomes during their lifecycle.

	Long slender form	Short stumpy form	Procyclic form
Stage	Proliferative	Quiescent	Proliferative
Surface coat	VSG	VSG	Procyclin (PE/GPEET)
Mitochondrium	Repressed	Repressed/enlarged	Active
Citric acid/respiratory chain enzymes	Absent	Present but not fully active	Present and activated
Energy source	D-Glucose	D-Glucose	L-Proline
Iron source	Tf, Lf, heme, and heme-containing proteins	Tf, Lf, heme, and heme-containing proteins	heme, heme-containing proteins
